# Supervised exercise training in patients with cancer during anthracycline-based chemotherapy to mitigate cardiotoxicity: a randomized-controlled-trial

**DOI:** 10.3389/fcvm.2023.1283153

**Published:** 2023-12-04

**Authors:** Caroline Schneider, Christoph Ryffel, Laura Stütz, Manuela Rabaglio, Thomas M. Suter, Kristin L. Campbell, Prisca Eser, Matthias Wilhelm

**Affiliations:** ^1^ Centre for Rehabilitation & Sports Medicine, Inselspital, Bern University Hospital, University of Bern, Bern, Switzerland; ^2^ Graduate School for Health Sciences, University of Bern, Bern, Switzerland; ^3^ Department of Cardiology, Inselspital, Bern University Hospital, Bern, Switzerland; ^4^ Department of Medical Oncology, Inselspital, Bern University Hospital, Bern, Switzerland; ^5^ Department of Physical Therapy, Faculty of Medicine, University of British Columbia, Vancouver, BC, Canada

**Keywords:** cardiotoxicity, global longitudinal strain, physical activity tracking, accelerometry, exercise training, biomarkers

## Abstract

**Background:**

Exercise training (ET) has been shown to mitigate cardiotoxicity of anthracycline-based chemotherapies (AC) in animal models. Data from randomized controlled trials in patients with cancer are sparse.

**Methods:**

Patients with breast cancer or lymphoma receiving AC were recruited from four cancer centres and randomly assigned to 3 months supervised ET. Primary outcome was change in left ventricular global longitudinal strain (GLS) from baseline (before AC) to post AC (AC-end) compared between the EXduringAC group, who participated in an exercise intervention during AC including the provision of an activity tracker, and the control group EXpostAC, who received an activity tracker only. Secondary outcome parameters were changes in high sensitivity Troponin T (hsTnT), NT-pro-brain natriuretic peptide (NT-proBNP), peak oxygen consumption (peak VO_2_) and objectively measured physical activity (PA) during this same time-period. All assessments were repeated at a 12-week follow-up from AC-end, when also the EXpostAC group had completed the ET, that started after AC. In exploratory analyses, robust linear models were performed to assess the association of PA with changes in echocardiographic parameters and biomarkers of LV function.

**Results:**

Fifty-seven patients (median age 47 years; 95% women) were randomized to EXduringAC (*n* = 28) and EXpostAC (*n* = 29) group. At AC-end, GLS deteriorated in both study groups (albeit insignificantly) with 7.4% and 1.0% in EXduringAC (*n* = 18) and EXpostAC (*n* = 18), respectively, and hsTnT and NT-proBNP significantly increased in both groups, without difference between groups for any parameter. Change in peak VO_2_ (−1.0 and −1.1 ml/kg/min) at AC-end was also similar between groups as was duration of moderate-to-vigorous PA (MVPA) with a median of 33 [26, 47] min/day and 32 [21, 59] min/day in the EXduringAC and EXpostAC group, respectively. In the robust linear model including the pooled patient population, MVPA was significantly associated with a more negative GLS and lesser increase in hsTnT at AC-end.

**Conclusion:**

In this small scale RCT, supervised ET during AC was not superior to wearing a PA tracker to mitigate cardiotoxicity. The dose-response relationship between PA and cardioprotective effects during AC found in our and previous data supports the notion that PA should be recommended to patients undergoing AC.

**Clinical Trial Registration:**

ClinicalTrials.gov, identifier NCT03850171.

## 1. Introduction

Due to improvements in anticancer therapies and early cancer detection, the number of cancer survivors has increased substantially over the last decades (1). There is a need for adequate long-term care of cancer survivors including the management of side-effects from cancer treatment. Anthracycline-based chemotherapies (AC) form an integral part of antineoplastic regimens in the treatment of breast cancer and lymphoma and have a dose-dependent adverse effect on the heart and/or vascular endothelial function (2). Although various mechanisms have been proposed to explain the AC-induced cardiovascular (CV) toxicity (3), the majority of studies suggest that an increase in oxidative stress, evidenced through the generation of reactive oxygen species and binding on topoisomerase-IIβ, may cause cardiac myocyte apoptosis, necrosis and damage to myocyte mitochondria (4, 5). The presence of shared risk factors (i.e., inactive lifestyle, obesity, smoking) (6) in conjunction with AC-induced CV toxicity further augment the risk of cardiovascular disease (CVD) in this population (7), and lead to a reduction in cardiorespiratory fitness (8). Indeed, a recent study reports that breast cancer survivors have a 1.8-fold increased risk of death from CVD 7 years post-diagnosis compared to age-matched women from the general population with CVD accounting for 35% of non-breast-cancer related mortality (9).

While left ventricular (LV) remodelling may not be apparent until months to years after chemo- and/or radiotherapy (10), other markers enabling the detection of subclinical cardiotoxicity have been proposed. These markers include a decrease in LV global longitudinal strain (GLS) (11) and elevation of biochemical markers of acute myocardial injury and haemodynamic stress, such as high-sensitivity Troponin-T (hsTnT) and N-terminal pro-brain natriuretic peptide (NT-proBNP), respectively (12).

Exercise-based cardio-oncology rehabilitation programmes have been recommended by the 2022 ESC Guidelines on cardio-oncology (12). Structured exercise training (ET) has been shown to prevent the loss in cardiorespiratory fitness (CRF), a global marker of cardiovascular health, in exercising patients (13–16) or prevent a further decline after AC in patients who sustained a decline in cardiac function (17).

Evidence for a cardioprotective effect of ET on cardiac markers during AC therapy stems mainly from animal studies which have been summarized in two meta-analyses, suggesting that ET improves fractional shortening, a marker of systolic function, in trained rats compared to their sedentary counterparts (18, 19). Both meta-analyses found greater cardioprotective effects when the ET programme was started prior to AC-therapy and conducted concomitantly to AC-therapy in comparison to after Doxorubicin exposure (18, 19).

In humans, data from cross-sectional and cohort studies assessing the association between self-reported physical activity (PA) and cardiac function support the hypothesis of a cardioprotective effect of exercise (20, 21). However, randomized controlled trials in humans are sparse, and results from animal studies could not be fully translated (15, 22, 23).

We therefore aimed to compare changes in markers of cardiac function and myocardial injury between patients performing supervised ET during AC and patients receiving only advice on PA, with all patients receiving an activity tracker. As many people nowadays use some form of PA tracking either with dedicated devices or simply by mobile phone apps, we used standardised PA tracking as the control intervention in order to account for the confounding influence of wearing a tracking device on levels of PA (24) and to provide objective PA measurements over 6 months in both groups. We hypothesized that ET during AC would mitigate the decline in GLS and increase in hsTnT and NTpro-BNP, compared to PA advice only. In exploratory analyses, we further aimed to assess the association of objectively measured PA with changes in GLS, LVEDVi, hsTnT and NTpro-BNP.

## 2. Materials and methods

### 2.1. Study design

The CAPRICE-Study (Cancer Adverse Effects Prevention with Care & Exercise) was a prospective, randomized controlled multicentre study, investigating the effect of exercise therapy timing with regard to cardiotoxicity and patient preference in early breast cancer and lymphoma patients receiving AC therapies (clinicaltrials.gov number NCT03850171). The study was conducted in four large Swiss cancer referral centres including one university hospital after it was approved by the Ethic Committee of the Canton of Bern, Switzerland.

Women and men aged 18 years and older with histologically confirmed breast cancer and lymphoma who were scheduled for first time AC were eligible for this study. Participants were excluded if they had: (1) a known or newly diagnosed structural heart disease; (2) a contraindication to maximal cardiopulmonary exercise testing (CPET); (3) cancer-specific contraindications to exercise; (4) previous radiotherapy of the mediastinum and/or the left breast; or (5) significant cognitive impairment or inability to follow the study procedures (i.e., due to language problems).

**Figure 1:**
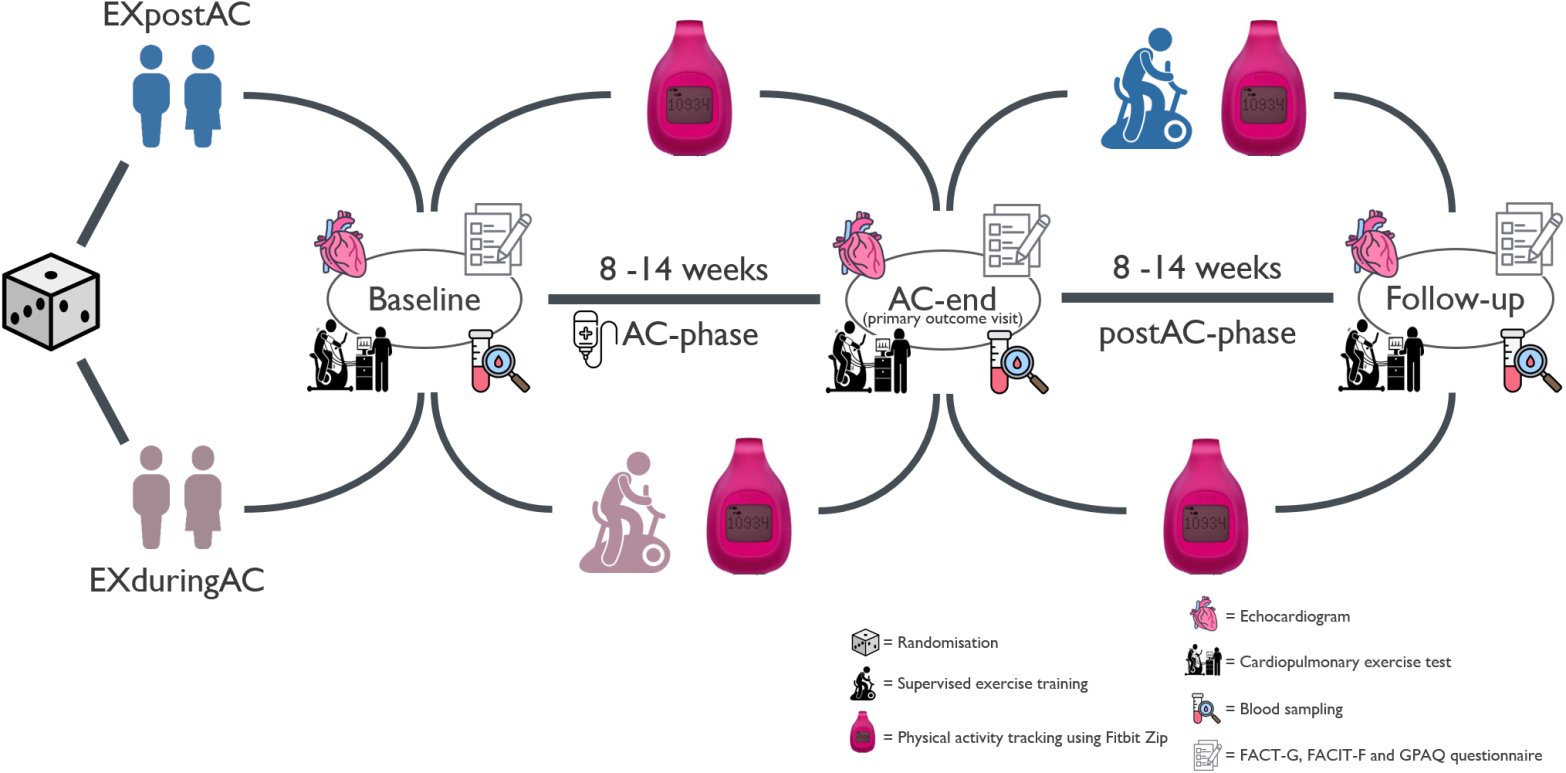
Study scheme. AC, anthracycline-based chemotherapies.

Group allocation was done using the minimization method, implemented in the randomization software MinimPy to balance between group characteristics as follows: type of cancer (breast cancer or lymphoma), age (< or ≥50 years, baseline left ventricular global longitudinal strain (≤−19% or >−19%), previous cancer therapy (previously treated with cardiotoxic therapy or not previously treated with cardiotoxic therapy), human epidermal growth factor receptor 2 (HER2) status (positive or negative) and chemotherapy cycle length (2 or 3 weeks). The allocation was performed after recruitment by an independent statistician from another university department. The intervention group participated in a centre-based cardio-oncologic rehabilitation programme during AC (EXduringAC), including the provision of an activity tracker, while the control group (EXpostAC) received an activity tracker only and performed the rehabilitation programme in the time-period from AC-end to 12-week follow-up. In the same time- frame from AC-end to 12-week follow-up, the EXduringAC group received an activity tracker only (Figure 1). Patients attended the laboratory for three visits: before or within 2 weeks of AC-initiation (baseline, week −1 to 2), in the first 2 weeks upon AC completion (AC-end, week 8–14, primary outcome visit) and at approximately 12 weeks after AC completion (12-week follow-up, week 20–26).

During all visits, patients provided a blood sample, completed an echocardiogram, a CPET for assessment of peak oxygen consumption (peak VO_2_) and a bio-impedance scan to obtain their body composition. Health-related quality of life (QoL) and fatigue were assessed using the validated functional assessment of chronical illness (FACIT) questionnaire, whereas physical activity pattern were evaluated with the global physical activity questionnaire (GPAQ). At the baseline visit, all patients were encouraged to perform leisure time PA according to current recommendations (25). There were no further instructions or follow-up calls with regard to PA. They received a hip- or bra-worn Fitbit Zip activity tracking device (Fitbit, San Francisco, USA) for the whole 6-month study duration and were instructed to wear it every day. In addition, all patients were advised to document the modality, duration and a subjective rating of exhaustion on the Borg Scale of all training sessions they completed in a training diary, including individual exercise performed at home and exercise as part of the rehabilitation programme. Breast cancer patients received 4 doses of AC either in a 14-day interval (dose-dense) or in a 21 day interval whereas lymphoma patients underwent 2–6 cycles of either R-CHOP or O-CHOP, escalated BEACOPP or ABVD-therapy with doxorubicin-administration every 3 weeks. A complete list depicting all administered AC is shown in the supplemental material (Supplementary Table 1).

In this paper we report the primary outcome change in GLS from baseline to AC-end and 12-week follow-up, and selected secondary outcome parameters at AC-end and 12-week follow-up (change in hsTnT, NT-proBNP, peak VO_2_ and PA). Detailed data on secondary outcomes (peak VO_2_, PA, quality of life and fatigue) will be reported elsewhere.

### 2.2. Supervised exercise training

Supervised ET was embedded in a 12-week ambulatory cardio-oncological rehabilitation programme, including 24 supervised centre-based and 12 non-supervised home-based exercise sessions, counselling on PA, psychological aspects, nutrition, cardiovascular risk factors and pain management. The ET was offered twice weekly to groups of approximately 10 cancer patients with exercise sessions lasting 90 min, supervised by experienced exercise therapists. Sessions started with 30 min of cycling on an ergometer (including a 5 min warm-up and 3 min cool-down) at a workload corresponding to the patient's individual first ventilatory threshold measured during the CPET (see below) of the baseline visit, increasing on a weekly basis targeting to achieve a score of 13 on the Borg scale (26) and duration of 40 min. After the cycling training, patients continued with approximately 40–45 min of strength (at 70%–80% of 1 repetition maximum, 2–3 sets of 8–12 repetitions for all major muscle groups, targeting a value of 15 on the Borg-Scale), coordination and/or balance training. At home, the Borg-Scale was recorded as a measure of intensity and the duration of the exercise.

#### 2.2.1. Control intervention

The control group received the Fitbit Zip device as did the intervention group and the simple verbal recommendation of performing a minimum of 150 min of at least moderate exercise per week.

### 2.3. Adaptations of supervised ET during the COVID-19-pandemic

During the Covid-19 restrictions, the rehabilitation programme was adapted to a hybrid-tele-rehabilitation model, comprising of one supervised individual ET session and two non-supervised ET sessions at home (27). Centre-based endurance sessions on the ergometer were partly replaced by Nordic Walking sessions outdoors. Home-based exercise sessions were supervised via the Fitbit Zip device and feedback was provided by the therapist via telephone or at the training centre, if possible. All patients were encouraged to perform at least 150 min of moderate PA per week, and two weekly strength sessions for major muscle groups (25).

### 2.4. Adherence with endurance exercise training

Adherence to the centre-based endurance training was defined as the percentage of completed training sessions relative to scheduled sessions. For patients in the EXduringAC group, adherence was calculated from the beginning of the ET intervention until the AC-end visit, whereas in the EXpostAC group, adherence was determined in the time-period from AC-end to 12-week follow-up from beginning to the end of ET participation, with 2 completed weekly centre-based sessions to achieve full adherence. In addition to compliance with solely centre-based ET, which for some patients had to be adapted due to Covid-19-related restrictions with only 1 supervised session per week, a total endurance adherence score was calculated, comprising centre-based and home-based exercise sessions. For full adherence 3 weekly exercise sessions had to be completed. For home-based trainings, only exercise sessions of at least 30 min and with ratings on the Borg Scale of 11 and higher were considered for analysis of adherence. Due to the group-based character of the centre-based exercise sessions, it was not possible to perform a detailed evaluation of the completed strength training (i.e., load, sets, and repetitions) in our cohort.

### 2.5. Transthoracic echocardiography

Standard transthoracic 2D echocardiography was performed before randomisation, at AC-end and at 12-week follow-up. All echocardiographic images were obtained on a Vivid 95 cardiac ultrasound system with a 7.5-MHz transducer (GE Medical system, New Jersey, U.S.A.) by experienced sonographers of an echo core lab using standard tomographic views. All data was stored on an external hard-drive and analysed off-line on a commercially available workstation using TomTec software (TomTec Imaging Software Systems, 2020). Traditional echocardiographic parameters of LV geometry, and systolic and diastolic function were assessed based on contemporary recommendations. Peak systolic LV GLS was assessed using standard 2D apical four-chamber, two-chamber and three-chamber views using speckle-tracking analysis (28). LV GLS was assessed by an experienced echocardiographer blinded for group allocation and time point.

### 2.6. Cardiopulmonary exercise testing

CPETs were performed at baseline, at AC-end and 12-week follow-up on a cycle ergometer with an individualized ramp protocol aiming to achieve exhaustion within 8–12 min. The protocol consisted of a 3-min warm-up at a workload of 5–20 watt followed by an increase of 10, 15, or 20 watt per minute until voluntary exhaustion and a 2-min active cool-down period. Throughout the CPET, patients were monitored by a cardiologist with continuous assessment of a 12-channel electrocardiogram. From May 2019 until the end of February 2020 gas exchange was measured using the breath-by-breath spirometry system Jaeger Oxycon Pro (Masterscreen CPX, PanGas Healthcare GmbH, Dagmersellen, Switzerland) which was thereafter replaced by the Quark spirometric system (Cosmed, Fehraltdorf, Switzerland). Peak VO_2_ was determined as the highest value of a 30 s moving average window. To include only valid peak VO_2_ in our analysis, we excluded CPETS with a respiratory exchange ratio below 1.05, since below this value the identified peak VO_2_ is from a submaximal test and does not indicate a true peak value.

### 2.7. Physical activity tracking

PA of all participants was tracked with a validated hip- or bra-worn Fitbit Zip device (29) from baseline to 12-week follow-up visit, which amounted to a duration of 6 months for most patients. The device enabled constant recording of PA parameters such as daily steps and daily minutes of low, medium and high activity. The Fitbit Zip was shown to be within the 10% equivalence zone around the indirect calorimetry estimate (30). Feedback regarding the daily steps could be seen by the patient on the screen of the device and greater detail on daily activity levels could be assessed by the patient on the Fitbit website. All patients were instructed to synchronize their devices at least once every week to ensure the data transfer into the Fitbit app on their phones, which was installed on the day of their first visit. At the AC-end and 12-week follow-up visits data was downloaded from the Fitibit Zip by the study team. Data from medium and high PA were sumarized to obtain one parameter, which we termed moderate to vigorous activity (MVPA). Additionally, all participants kept a training diary in which they documented modality, duration and Borg Scale of all training sessions they completed, including individual exercise performed at home and exercise as part of the rehabilitation programme. Training sessions not reflected by the Fitbit Zip, such as cycling or swimming, or when the Fitbit Zip device was not worn, were added to the Fitbit Zip PA and step data using a formula (if their duration was at least 10 min and Borg scale ≥12, see Supplementary Appendix). A detailed description of the following sections of the methods can be found in the Supplements: Transthoracic echocardiography, Biomarkers of myocardial injury, Physical activity tracking, and Serious adverse events.

### 2.8. Sample size calculation

Sample size calculation was based on previous studies that measured GLS before and after AC in patients with breast cancer (31, 32). Based on these studies, we assumed that the EXpostAC group will increase GLS by 2% from −20% to −18% and the EXduringAC group will have an increase of only 0.5% from −20% to −19.5% (with a standard deviation of 2.5%), congruent with a clinically meaningful difference between the two groups (33). Based on a one-sided t-test, an alpha of 0.05 and power of 0.8, 102 patients (51 patients per group) were required, assuming a 15% dropout rate.

### 2.9. Data analysis

We used the R Studio software (Version 2022.02.3 + 492, R version 4.2.1.) for all statistical analyses.

Patient characteristics were compared between groups using Wilcoxon rank sum tests, Chi-square or Fisher's exact test, as appropriate. Statistical significance was set at alpha <0.05.

The effect of the exercise intervention on changes in markers of LV function, myocardial injury and peak VO_2_ were compared between the two groups by linear mixed model with group (EXduringAC and EXpostAC) and time point (visits) as fixed factors including their interaction. Patients were included as random intercepts. Primary outcome was the group × time interaction for GLS from baseline to AC-end of patients with available GLS at both time points. An additional model was performed for GLS with heart rate as a covariate to adjust for higher heart rate that can arise as a consequence of AC-induced anaemia and consequent changes in preload. Further, a second model was performed for peak VO_2_, adjusted for age and BMI. Due to assay-specific non-detection values of <5 ng/L and <50 pg/ml for hsTnT and NT-proBNP, respectively, a conservative approach was used by setting these values at 4 ng/L for hsTnT and at 49 pg/ml for NT-proBNP. Log transformation was used when necessary.

In exploratory analyses, robust linear models were performed for change in GLS, LVEDVi, hsTnT and NT-proBNP from baseline to AC-end for the pooled patient population, including treatment group and objectively measured PA (expressed as daily MVPA or steps) as predictor variables.

All analyses were performed on an intention-to-treat (ITT) basis. In addition, per protocol (PP) analyses were performed. PP was defined according to which phase (AC or post-AC phase) patients performed at least 60% of the centre-based training sessions in. Patients who did not complete 60% of the planned training sessions in neither phase were excluded from the PP analysis. Missing data was not imputed.

## 3. Results

### 3.1. Study flow and baseline characteristics

**Figure 2:**
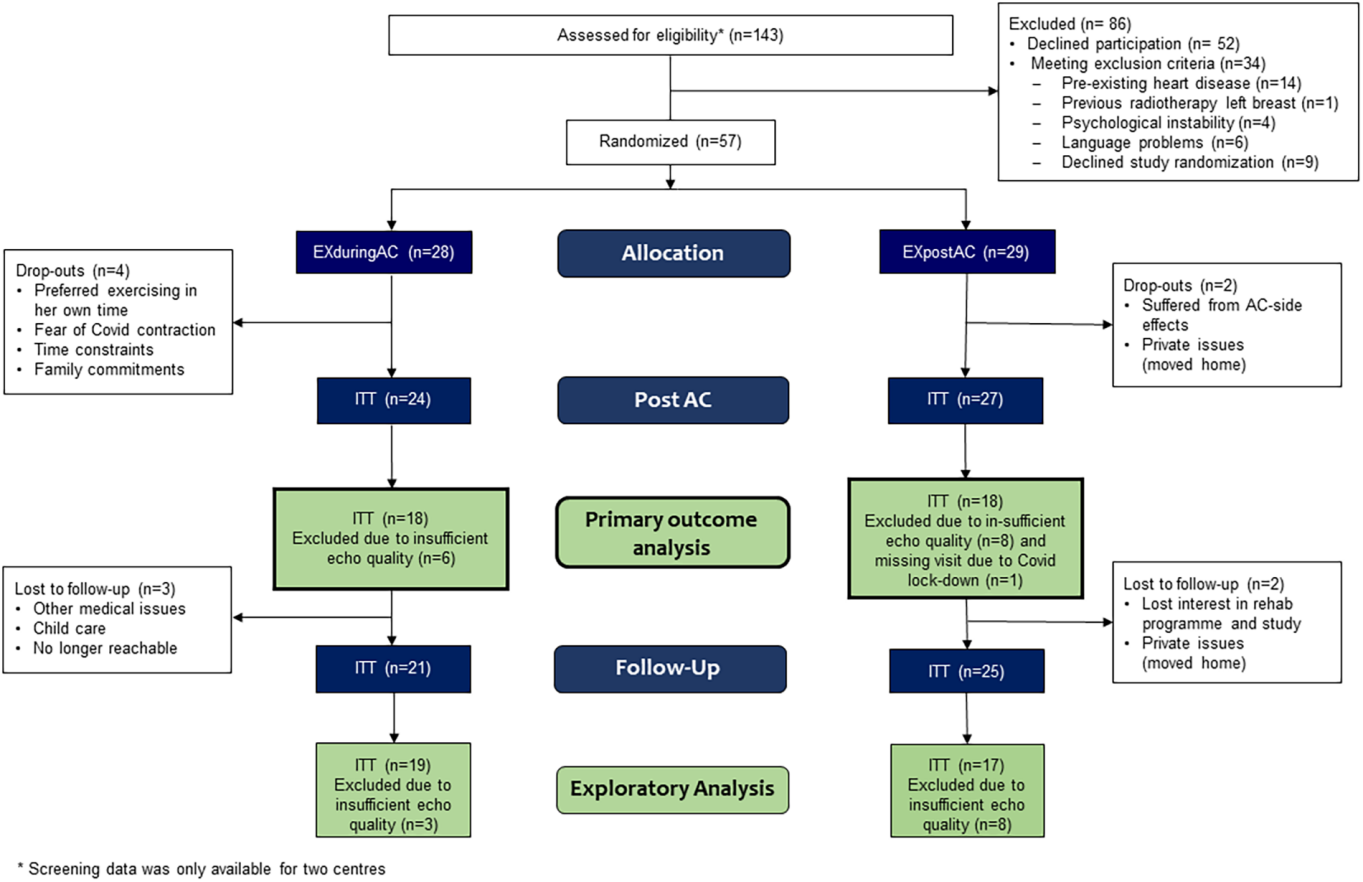
Study flow.

The patient flow is shown in Figure 2 (and for PP-analysis in Supplementary Figure 1). Between May 2019 and June 2022, 143 patients were assessed for eligibility at four centres (whereby two small centres supplied incomplete screening lists), and 57 patients consented to participate in the study and were randomized. Twenty-eight patients were allocated to EXduringAC and 29 to EXpostAC. As a consequence of slow recruitment due to Covid-19 and limited funding, the study was stopped before the target sample size was reached. Baseline demographic and clinical characteristics are shown in Table 1.

**Table 1:** Baseline characteristics of the study population, shown are *n* (%), or median [1st and 3rd quartiles].

Characteristics	All participants (*n* = 57)	EXduringAC (*n* = 28)	EXpostAC (*n* = 29)	*p*-value
Inclusion site				
Centre 1 (University hospital)	23	12	11	
Centre 2	23	11	12	
Centre 3	6	3	3	
Centre 4	5	2	3	
Male	4 (7.0)	2 (7.1)	2 (6.8)	1
Age [years]	47 [38, 57]	50 [38, 57]	46 [38, 57]	0.798
Body mass index [kg/m^2^]	23.9 [21.6, 27.1]	23.0 [21.9, 26.4]	24.1 [21.5, 27.8]	0.497
Systolic blood pressure [mmHg]	115 [110, 120]	116 [112, 120]	114 [106, 120]	0.249
Diastolic blood pressure [mmHg]	70 [65, 78]	69 [65, 78]	70 [62, 78]	0.979
Hemoglobin [g/L]^a^	12.9 [12.1, 13.9]	13.0 [12.3, 13.9]	12.9 [12.0, 13.8]	0.966
Anaemia^a^	15 (28.8)	8 (28.6)	7 (24.1)	0.836
Cardiorespiratory fitness [ml/min/kg]	24.9 [21.2, 29.2]	24.2 [21.6, 28.4]	25.8 [21.2, 29.9]	0.794
Cardiorespiratory fitness [% of predicted by Wasserman]	103 [88, 118]	101 [88, 118]	105 [84, 116]	0.854
Cardiorespiratory fitness [% of predicted by FRIEND]	88.8 [75.6, 101.0]	86.7 [76.1, 102.6]	94.6 [72.7, 100.8]	0.629
GPAQ MVPA (min/week)	440 [270, 734]	531 [337, 791]	369 [237, 694]	0.348
Tumor site				
Breast	52 (91.2)	26 (92.9)	26 (89.7)	0.9999
Lymphoma	5 (8.8)	2 (7.1)	3 (10.3)	
BC disease-stage (I, II, III, IV)				
Stage I	12 (23.1)	7 (26.9)	5 (19.2)	0.679
Stage II	30 (57.7)	13 (50.0)	17 (65.5)	
Stage III	9 (17.3)	5 (19.2)	4 (15.4)	
Stage IV	1 (1.9)	1 (3.8)	0 (0.0)	
BC clinical subtype				
Hormone-positive (ER+/PR+)	30 (52.6)	13 (50.0)	17 (65.4)	0.400
HER2-positive	14 (26.9)	6 (23.1)	8 (30.8)	0.755
Triple negative	13 (25.0)	7 (26.9)	6 (23.1)	1
AC (only for BC)				
Neoadjuvant (with preceeding chemotherapy	10 (17.5)	5 (17.9)	5 (17.2)	0.932
Neoadjuvant (no preceeding chemotherapy)	16 (28.1)	7 (25.0)	9 (31.0)	
Adjuvant	26 (45.6)	14 (50.0)	12 (41.4)	
Dose-dense	25 (43.9)	11 (39.3)	14 (48.3)	0.452
Cumulative AC-dose				
BC	236.8 [231.2, 240.0]	240 [234.4, 242.0]	233.5 [228.3, 238.5]	0.021
Lymphoma	293.0 [140.0, 300]	200.0 [150.0, 250.0]	293.0 [216.5, 296.5]	1
Other cancer therapy				
Herceptin	11 (19.3)	4 (14.3)	7 (24.1)	0.747
Radiotherapy	39 (68.4)	19 (67.9)	20 (69.0)	1
Left-sided	16 (28.1)	9 (32.1)	7 (24.1)	0.751
Taxol	38 (66.7)	18 (64.3)	20 (69.0)	0.893
Hormone therapy	14 (26.9)	7 (25.0)	7 (24.1)	1
Smoking				
Current	4 (7.0)	2 (7.1)	2 (6.9)	0.9999
Former (more than 3 months)	12 (21.1)	7 (25.0)	5 (17.2)	
Cardiovascular risk factors				
Diabetes mellitus	2 (3.5)	0 (0.0)	2 (6.9)	0.491
Hyperlipidemia	9 (15.8)	4 (14.3)	5 (17.2)	1
Obesity	6 (10.5)	1 (3.6)	5 (17.2)	0.194
Hypertension	3 (5.3)	0 (0.0)	3 (10.3)	0.237
Sum score cardiovascular risk factors	0 [0, 1]	1 [0, 1]	0 [0, 1]	0.077

Indicated are *p*-values from Wilcoxon two-sample tests or Fisher's exact test, as appropriate.

BC, breast cancer; AC, anthracycline, GPAQ, global physical activity questionnaire; MET, metabolic equivalent.

^a^ Anaemia was defined as hemoglobin level <120 g/L for women and <130 g/L for men.

Six patients (10.5%) discontinued the study before the AC-end visit (4 EXduringAC, 2 EXpostAC). Patients who dropped out of the study were 11 years older compared to the remaining study cohort (*p* = 0.007) but did not differ with regard to other baseline characteristics (Supplementary Table 2). Reasons for discontinuation of the study were time constraints due to private commitments and other medical appointments, side-effects from AC-therapy, or no longer being interested in the study. Six patients chose the timing of their rehabilitation participation discordant with their randomised group allocation. Twenty and 17 patients fulfilled the PP criteria in the EXduringAC and EXpostAC group, respectively. However, results of all models were consistent between ITT and PP analyses, so the effect of non-compliant patients or patients changing group was negligible. Due to insufficient image quality at baseline and/or at AC-end and a missing visit related to Covid-19, primary outcome data of GLS could not be analysed in 15 patients.

Ninety-one percent of the participants had breast cancer and 9% had lymphoma. The majority of patients with breast cancer were diagnosed with HER2+ (53%) and 50% of breast cancer patients were undergoing neoadjuvant AC. Three patients (2 EXduringAC, 1 EXpostAC) received 4 cycles of Carboplatin and 6 patients (4 EXpostAC, 2 EXduringAC-group) received 4 cycles of Trastuzumab prior to AC administration.

AC for all but one breast cancer participants consisted of 4 cycles with 60 mg/m^2^ doxorubicin combined with 600 mg/m^2^ cyclophosphamide. One patient not receiving doxorubicin was administered an equivalent dose of epirubicin. Median [1st and 3rd quartiles] cumulative dose in the EXduringAC group and EXpostAC group was 240 mg/m^2^ [234.4, 242.0 mg/m^2^] and 233.5 mg/m^2^ [228.3, 238.5 mg/m^2^], respectively, which was a small but significant difference between groups.

Two lymphoma patients were diagnosed with Hodgkin's lymphoma (stages II and III), two patients suffered from diffuse large B-cell-lymphoma (stages II and III) and one patient was diagnosed with follicular lymphoma (stage III–IV). Three lymphoma patients received six cycles of either R-CHOP or O-CHOP-therapy, 1 patient received 4 cycles of escalated BEACOPP and 1 patient was treated with 2 cycles of ABVD-therapy. All participants were administered the prescribed AC-dose with no dose reductions occurring.

In one patient randomized to the EXduringAC group, LVEF dropped from 55% at baseline to 39% at 12-week follow-up (−29%), which we documented as a serious adverse event. After evaluation with the data safety monitoring committee of the study, it was concluded that a causal relation of the event with the study intervention was unlikely. The patient received treatment with an angiotensin-converting-enzyme inhibitor and beta-blocker and was thereafter regularly monitored in the cardio-oncology-unit of the University Hospital. Due to insufficient image quality at baseline, this patient was excluded from further analyses of our primary outcome.

### 3.2. Intervention adherence

For patients completing ET during AC, ET was started on a median [1st and 3rd quartiles] of 9 days (7, 11) after their first AC-cycle, whereas for patients completing the ET after AC, ET was initiated on a median [1st and 3rd quartiles] of 28 days (19, 45) after their final AC-cycle. According to ITT-analysis, median [1st, 3rd quartile] adherence with the centre-based endurance training sessions from initiation of ET until the AC-end visit in the EXduringAC group was 65% [36, 78], whereas adherence from beginning of ET until the 12-week follow-up visit was 71% [38, 88] in the EXpostAC group. Of 19 and 18 patients in the EXduringAC group and the EXpostAC group, respectively, with complete documentation on PA, total adherence with endurance exercise training, including centre-and home-based exercise sessions was 71% [59, 93] and 87% [67, 100], respectively.

Covid-19-related adaptations of the centre-based exercise sessions required the shortening of exercise sessions to 60 min in some patients with the consequence that due to time-restrictions, endurance exercise sessions were replaced with strength training, which contributed to the low adherence with the centre-based ET.

### 3.3. Changes in echocardiographic parameters and biomarkers of subclinical cardiotoxicity

Baseline data and changes in echocardiographic parameters are shown in Table 2 for ITT groups (Supplementary Table 3 for PP groups). Individual changes in GLS, LVEDVi, hsTnT and NT-proBNP are shown in Supplementary Figure 2. At baseline, echocardiographic and biomarkers were not significantly different between groups. Changes from baseline to AC-end and 12-week follow-up in echocardiographic markers were similar with no differences between the two study groups and consistent results in the ITT (Tables 2 and 3) and PP analyses (Supplementary Table 3).

**Table 2:** Baseline values and changes of echocardiographic parameters and biomarkers of myocardial injury according to allocated study group (Intention-to-treat analysis).

	EXduringAC (*n* = 28)			EXpostAC (*n* = 29)		
	Baseline	Δ baseline to AC-end	Δ AC-end to follow-up	Baseline	Δ baseline to AC-end	Δ AC-end to follow-up
GLS [%]	−21.9 (−22.8, −19.1)	−1.05 (−3.0, 3.1)	0.6 (−1.1, 2.7)	−21.1 (−22.8, −19.1)	0.35 (−1.8, 2.6)	−0.9 (−3.0, 3.8)
	*N* = 24	*N* = 18	*N* = 19	*N* = 24	*N* = 18	*N* = 17
HR GLS [bpm]	71.5 (67.0, 79.8)	0.5^a^ (−5.8, 12.3)	−5.0 (−8.8, 1.5)	67.0 (62.5, 76.3)	8.5^a^ (3.0, 14.3)	−3.5 (−8.0, 2.5)
	*N* = 24	*N* = 18	*N* = 18	*N* = 24	*N* = 18	*N* = 16
2D LVEF [%]	64.5 (57.7, 68.3)	−1.5 (−5.7, 3.4)^a^	0.2 (−5.9, 4.4)	63.9 (61.4, 67.9)	−3.5 (−6.0, 0.3)^a^	2.0 (−3.9, 4.0)
	*N* = 26	*N* = 22	*N* = 21	*N* = 29	*N* = 25	*N* = 23
LVEDVi [ml/m^2^]	52.3 (43.8, 60.6)	−2.6 (−6.6, 4.4)	−5.8 (−10.2, 7.9)	47.3 (39.0, 53.1)	0.8 (−9.5, 7.7)	−0.4 (−13.1, 6.2)
	*N* = 25	*N* = 21	*N* = 21	*N* = 26	*N* = 24	*N* = 23
*E*/*e*’	6.6 (6.1, 8.1)	−0.04 (−1.55, 0.91)	0.47 (−1.37, 1.00)	7.2 (5.7, 8.1)	−0.36 (−0.78, 1.49)	0.00 (−0.90, 0.84)
	*N* = 28	*N* = 24	*N* = 22	*N* = 29	*N* = 26	*N* = 23
*E*/*A* ratio	1.12 (1.0, 1.37)	−0.09 (−0.22, 0.08)	0.00 (−0.20, 0.24)	1.25 (0.93, 1.44)	−0.09 (−0.14, 0.20)	−0.06 (−0.37, 0.12)
	*N* = 28	*N* = 25	*N* = 23	*N* = 29	*N* = 26	*N* = 24
LAVI [ml/m^2^]	22.6 (19.9, 28.4)	−2.7 (−4.6, 3.11)	0.3 (−3.2, 5.9)	20.6 (18.9, 26.7)	0.4 (−1.3, 3.7)	−0.9 (−4.3, 2.2)
	*N* = 26	*N* = 22	*N* = 21	*N* = 29	*N* = 23	*N* = 21
NT-proBNP [pg/ml]	56.5 (49.0, 82.0)	35.0 (0.0, 107.0)^a^	−1.0 (−71.0, 41.0)	49.0 (49.0, 53.3)	41.0 (0.0, 93.0)^a^	−37.0 (−107.0, 0.0)
	*N* = 28	*N* = 21	*N* = 19	*N* = 28	*N* = 23	*N* = 21
hsTnT [ng/L]	4.0 (4.0, 4,0)	8.0 (6.0, 16.0)^a^	−2.0 (−10.0, 1.0)^a^	4.0 (4.0, 4,0)	10.0 (6.3, 14.8)^a^	−5.0 (−9.0, −1.0)^a^
	*N* = 25	*N* = 21	*N* = 18	*N* = 25	*N* = 22	*N* = 21
hs-C-reactive protein [mg/L]	1.03 (0.77, 1.58)	0.98 (−0.06, 2.49)	−0.96 (−2.63, 0.29)	1.91 (0.97, 3.68)	0.81 (−0.02, 1.37)	−0.56 (−2.83, 0.73)
	*N* = 25	*N* = 22	*N* = 18	*N* = 26	*N* = 22	*N* = 22
Systolic BP [mmHg]	122.5 (116.0, 134.5)	−5.5 (−13.0, 10.0)	1.0 (−10.0, 15.0)	120.0 (111.5, 135.5)	0.5 (−8.8, 14.8)	−2.0 (−5.0, 5.5)
	*N* = 26	*N* = 20	*N* = 17	*N* = 27	*N* = 22	*N* = 20
Diastolic BP [mmHg]	75.0 (65.8, 84.8)	−2.0 (−10.3, 7.0)	−3.0 (−13.0, 12.0)	74.0 (63.0, 80.0)	−1.5 (−9.5, 11.3)	1.0 (−6.3, 9.3)
	*N* = 26	*N* = 20	*N* = 17	*N* = 27	*N* = 22	*N* = 20

Shown are median (1st and 3rd quartiles). Number of patients with sufficient echo quality are also indicated. Significant *p*-values for group and time interaction or main effects are indicated and derived from mixed linear models with patients as random factors (intercepts) and EXpostAC group and baseline time point as references.

GLS, global longitudinal strain; HR, heart rate; LVEF, left ventricular ejection fraction; LVEDVi, left-ventricular end-diastolic volume index; hsTnT, high-sensitivity troponin-T; NT-proBNP, N-terminal pro-brain natriuretic peptide; LAVI, left atrial end-systolic volume index; hs-C-reactive protein, high-sensitivity C-reactive protein; BP, blood pressure.

^a^ *p* < 0.05 for main time effect.

**Table 3:** Mixed linear models with fixed effects group and time point (including interaction) and patients as random factors (intercept).

	Estimate (95% CI)		*t*-value	*p*-value
GLS				
Intercept	−21.08	(−22.32,−19.84)	−33.78	0.000
EXduringAC	0.45	(−1.31, 2.21)	0.52	0.608
AC-end	0.63	(−0.89, 2.15)	0.83	0.410
Follow-up	0.83	(−0.63, 2.29)	1.14	0.258
EXduringAC × Visit 2	−0.14	(−2.26, 1.98)	−0.14	0.892
EXduringAC × Visit 3	0.92	(−1.18, 3.02)	0.88	0.382
LVEDVi				
Intercept	46.77	(41.89, 51.65)	19.20	0.000
EXduringAC	4.95	(−1.99, 11.89)	1.43	0.159
AC-end	0.20	(−4.98, 5.38)	0.08	0.938
Follow-up	−0.74	(−6.06, 4.58)	−0.28	0.781
EXduringAC × Visit 2	−0.42	(−7.92, 7.08)	−0.11	0.910
EXduringAC × Visit 3	0.80	(−6.98, 8.58)	0.20	0.839
Log (hsTnT)				
Intercept	1.42	(1.24, 1.6)	16.49	0.000
EXduringAC	−0.01	(−0.25, 0.23)	−0.05	0.961
AC-end	1.21	(0.99, 1.43)	10.68	**0.000**
Follow-up	0.76	(0.52, 1)	6.63	**0.000**
EXduringAC × Visit 2	0.01	(−0.31, 0.33)	0.06	0.954
EXduringAC × Visit 3	0.19	(−0.15, 0.53)	1.12	0.268
Log (NT-proBNP)				
Intercept	4.03	(3.81, 4.25)	35.62	0.000
EXduringAC	0.13	(−0.19, 0.45)	0.78	0.438
AC-end	0.57	(0.31, 0.83)	4.21	**0.000**
Follow-up	0.17	(−0.11, 0.45)	1.23	0.223
EXduringAC × Visit 2	−0.13	(−0.51, 0.25)	−0.66	0.514
EXduringAC × Visit 3	0.20	(−0.2, 0.6)	0.98	0.332

Reference category was EXpostAC and baseline. Analysis was done according to Intention-to-treat. GLS, global longitudinal strain; LVEDVi, left-ventricular end-diastolic volume index; hsTnT, high-sensitivity Troponin-T; NT-proBNP, N-terminal pro-brain natriuretic peptide.

Significant values are indicated in bold.

GLS at baseline was within the age-expected range (Table 2).

In patients with available GLS at baseline and AC-end (*n* = 18 in both groups), GLS at baseline was −21.6% [−22.3, −19.0%] and −21.0% [−22.5, −19.0%] in the EXduringAC group and EXpostAC group respectively. At AC-end, GLS was −20.0% [−22.7, −18.8%] and −20.8% [−23.8, −16.9%] in the EXduringAC group (change of 7.4% compared to baseline) and EXpostAC group (change of 1.0% compared to baseline), respectively.

**Figure 3:**
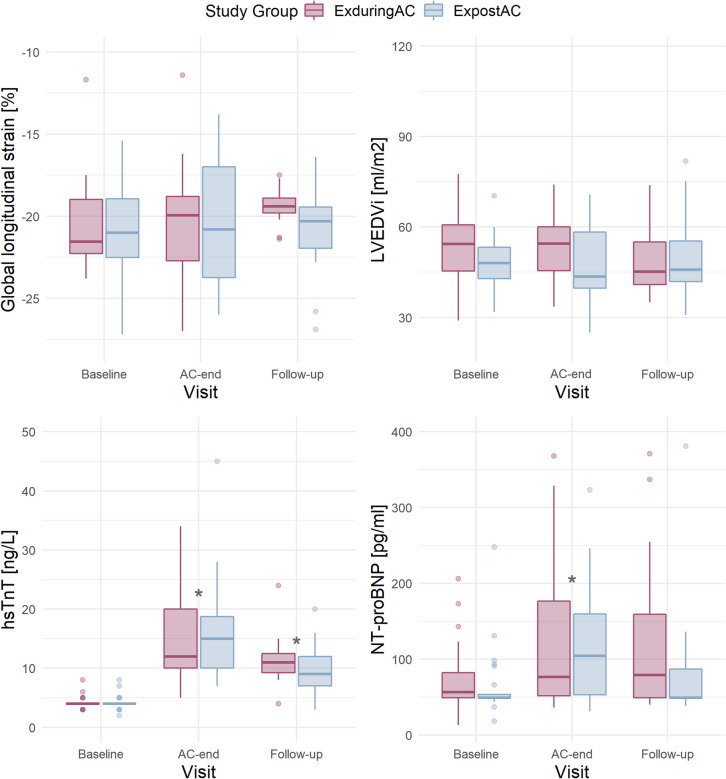
Boxplots per study group (according to Intention-to-treat) and visit in patients with available data at baseline and AC-end for GLS (*n* = 18 in the EXduringAC and *n* = 17 in the EXpostAC group), LVEDVi (*n* = 21 and *n* = 24), hsTnT (*n* = 21 and *n* = 22) and NT-proBNP (*n* = 24 and *n* = 25). AC, anthracycline-based chemotherapies; GLS, global longitudinal strain; LVEDVi, left-ventricular end-diastolic volume index; hsTnT, high-sensitivity troponin-T; NT-proBNP, N-terminal pro-brain natriuretic peptide.

At 12-week follow-up and in patients with complete GLS data at baseline and AC-end (*n* = 18 in EXduringAC, *n* = 18 in EXpostAC), GLS was −19.40 [−19.8, −18.9] in the EXduringAC group and −20.30 [−22.0, −19.5] in the EXpostAC group (Figure 3).

When analysed over the entire study groups, change in GLS did not exceed the threshold of −15% that has been suggested to predict a future drop in LVEF (at no time-point) (34). Individual changes are depicted in Supplementary Figure 2. In an additional linear mixed model adjusted for heart rate, a higher heart rate was associated with a less negative GLS, whereby an increase in heart rate of 10/min resulted in an 0.8% change in GLS (Supplementary Table 4).

At AC-end, there was a significant increase in hsTnT by a median of 8.0 ng/L [6.0, 16.0 ng/L] and 10.0 ng/L [6.3, 14.8 ng/L] in the EXduringAC group and EXpostAC group, respectively (*p* < 0.05 in both groups). NT-proBNP also increased significantly by a median of 35.0 pg/ml [0.0, 107.0 pg/ml] and 41.0 pg/ml [0.0, 93.0 pg/ml] in the EXduringAC group and EXpostAC group, respectively (*p* < 0.05 in both groups), with no changes between the groups (*p* for interaction group × time = 0.954 and 0.514 for hsTnT and NT-proBNP, respectively). The increase in hsTnT persisted at follow-up, whereas NT-proBNP was no longer different when compared to baseline (Tables 2 and 3, Figure 3).

### 3.4. Changes in cardiorespiratory fitness

Peak VO_2_ tended to decrease from the baseline to the AC-end visit from a median 24.2 ml/kg/min [1st and 3rd quartile 21.6, 28.4 ml/kg/min] to 23.2 ml/kg/min [20.9, 27.0 ml/kg/min] in the EXduringAC and from 25.8 ml/kg/min [21.2, 29.9 ml/kg/min] to 24.7 ml/kg/min [19.8, 27.7 ml/kg/min] in the EXpostAC group. From AC-end to 12-week follow-up peak VO_2_ increased (albeit insignificantly) to 25.1 ml/kg/min [20.1, 27.7 ml/kg/min] and 26.7 ml/kg/min [22.9, 32.4 ml/kg/min]in the EXduringAC group and EXpostAC group, respectively. There was no significant group × time interaction indicating that there was no treatment effect for peak VO_2_ neither at AC-end, nor 12-week follow-up (*p* = 0.801 and *p* = 0.413, respectively). Detailed results on peak VO_2_ and PA data will be published elsewhere.

### 3.5. Physical activity data

Self-reported PA at baseline achieved recommended levels of guidelines in both groups (Table 1). At AC-end, PA data recorded by Fitbit Zip and complemented with data from training diaries for cycling and swimming was available from 43 patients. Detailed PA data will be presented elsewhere. Daily duration of PA did not differ between EXduringAC and EXafterAC, and neither did daily number of steps. MVPA was a median 33 min/day [26, 47] min/day and 32 min/day [21, 59] min/day in the EXduringAC and EXpostAC group, respectively, and for steps a median 7,641 [6,332, 9,810] steps/day and 6,806 [5,275, 9,429] steps/day, respectively. Overall, 76% of our cohort reached the recommended amount of MVPA of 150 min/week during AC (Central Figure).

**CENTRAL FIGURE:**
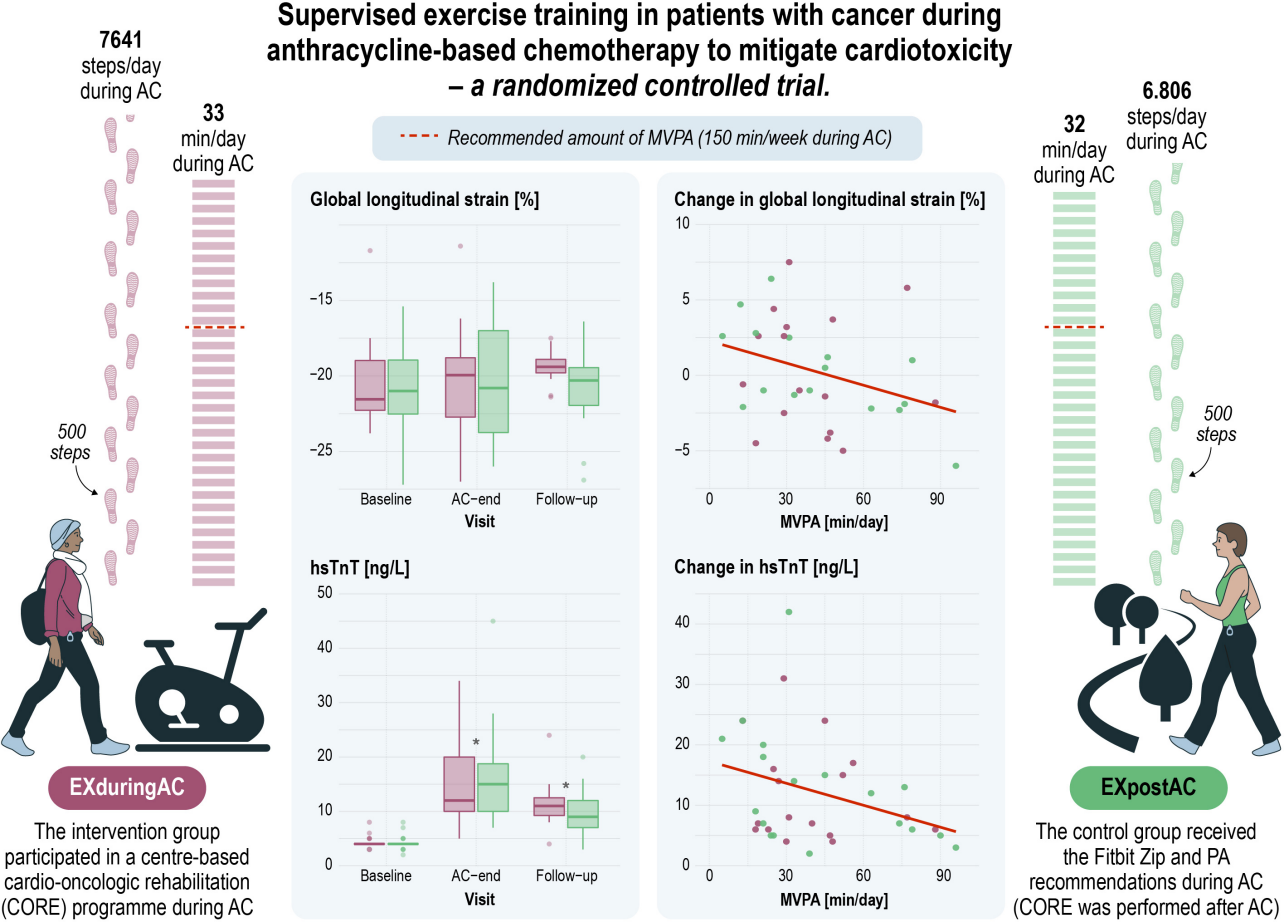
Supervised exercise training in patients with cancer after anthracycline-based chemotherapy to mitigate cardiotoxicity—a randomised controlled trial. AC, anthracycline-based chemotherapy; hsTnT, high-sensitivity troponin T; MVPA, moderate and vigorous physical activity.

### 3.6. Association of physical activity with markers of LV dysfunction and myocardial injury at AC-end

**Figure 4:**
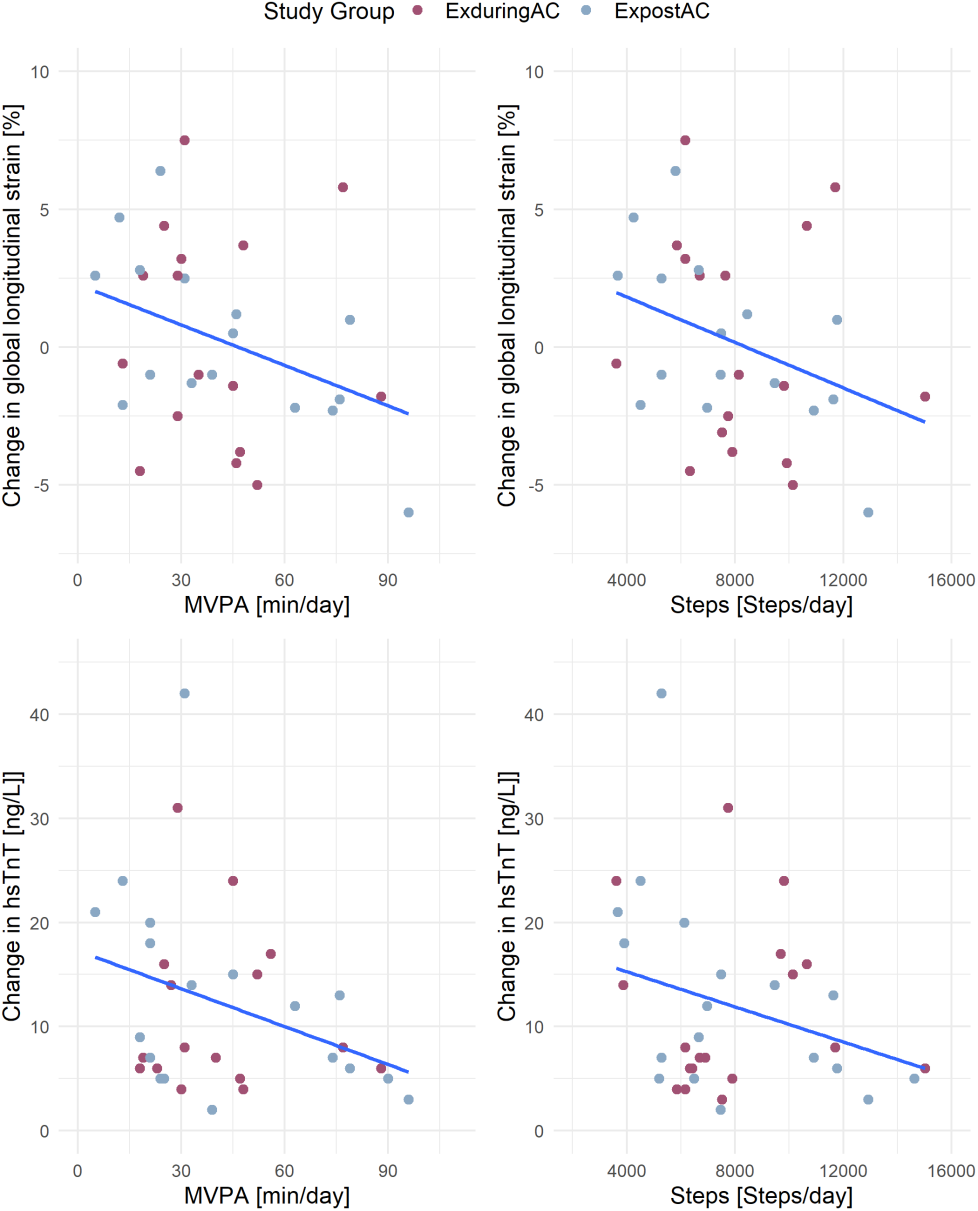
Association of activity parameters (MVPA and steps) with changes in GLS and hsTnT. MVPA, moderate to vigorous physical activity; GLS, global longitudinal strain; hsTnT, high-sensitivity troponin-T.

In the linear models including the pooled patient population, a higher step count was associated with a more negative GLS, with an increase of 2,000 steps/day corresponding to a 1% more negative GLS. A similar association was found for MVPA with GLS and hsTnT, with a 30 min increase in MVPA corresponding to a 1.8% more negative GLS and a 3 ng/L lesser increase in hsTnT. We did not find any associations between activity parameters and LVEDVi or NT-proBNP (Figure 4 and Table 4).

**Table 4:** Robust linear models for changes in GLS, LVEDVi, hsTnT and NT-proBNP from baseline to AC-end, including study group and activity parameters steps (left column) or MVPA (right column) as predictors.

	Estimate (95% CI)		*t*-value	*p*-value		Estimate (95% CI)		t-value	*p*-value
GLS					GLS				
Intercept	3.78	(−0.22, 7.78)	1.89	0.069	Intercept	2.41	(−0.45, 5.27)	1.69	0.104
EXduringAC	0.18	(−2.28, 2.64)	0.15	0.885	EXduringAC	0.62	(−1.78, 3.02)	0.51	0.611
HR GLS	0.07	(−0.09, 0.23)	0.83	0.412	HR GLS	0.02	(−0.1, 0.14)	0.32	0.755
Steps	0.00	(0, 0)	−2.36	**0.026**	MVPA	−0.06	(−0.12, 0)	−2.30	**0.030**
*R*^2^: 11%					*R*^2^: 10%				
LVEDVi					LVEDVi				
Intercept	−0.28	(−10.92, 10.36)	−0.05	0.958	Intercept	−2.44	(−11.46, 6.58)	−0.54	0.592
EXduringAC	−0.63	(−8.47, 7.21)	−0.16	0.873	EXduringAC	0.05	(−7.83, 7.93)	0.01	0.989
Steps	0.00	(0,0)	0.04	0.971	MVPA	0.06	(−0.1, 0.22)	0.73	0.471
*R*^2^: 5%					*R*^2^: 4%				
hsTnT					hsTnT				
Intercept	19.21	(10.47, 27.95)	4.40	0.000	Intercept	15.65	(8.51, 22.79)	4.38	0.000
EXduringAC	−1.19	(−7.07, 4.69)	−0.41	0.688	EXduringAC	−0.44	(−5.54, 4.66)	−0.18	0.863
Steps	0.00	(0,0)	−1.71	0.097	MVPA	−0.10	(−0.18, −0.02)	−2.36	**0.025**
*R*^2^: 3%					*R*^2^: 6%				
NT-proBNP					NT-proBNP				
Intercept	85.57	(28.35, 142.79)	2.99	0.005	Intercept	69.34	(25.04, 113.64)	3.13	0.004
EXduringAC	6.18	(−47.28, 59.64)	0.23	0.819	EXduringAC	8.53	(−49.47, 66.53)	0.29	0.770
Steps	−0.01	(−0.01, −0.01)	−1.67	0.104	MVPA	−0.55	(−1.31, 0.21)	−1.43	0.163
*R*^2^: 1%					*R*^2^: 2%				

For GLS, models were adjusted for heart rate. Reference category was EXpostAC. Group allocation was according to Intention-to-treat. In the models using total steps and MVPA as predictor, the final models were based on 28 and 27 patients for GLS, 36 and 35 patients for LVEDVi, 33 and 32 patients for hsTnT, and 34 and 33 patients for NT-proBNP, for EXduringAC and EXpostAC, respectively. The percentages of total variance explained by the models is shown with the *R*^2^-value. GLS, global longitudinal strain; MVPA, moderate to vigorous physical activity; HR, heart rate; LVEDVi, left-ventricular end-diastolic volume index; hsTnT, high-sensitivity troponin-T; NT-proBNP, N-terminal pro-brain natriuretic peptide; CI, confidence interval; *R*^2^, adjusted *R*^2^-value.

Significant values are indicated in bold.

## 4. Discussion

In this small scale randomized controlled trial in patients with cancer receiving AC, the addition of supervised ET during or after AC was not superior to PA tracking alone to mitigate cardiotoxicity. Changes in GLS, hsTnT and NT-proBNP did not differ between the EXduringAC and EXpostAC groups at neither time-point. Importantly, PA at baseline and objectively measured PA at AC-end achieved recommended levels of guidelines in both groups. In our active cohort, supervised ET during AC did neither lead to an increase in PA nor to a lesser decrease in peak VO_2_ compared to the control intervention of PA tracking only. In the pooled patient population we found a significant inverse association between PA and changes in GLS and hsTnT at AC-end. PA tracking is common practice nowadays and the potential of PA tracking as alternative to enrolling in an ET programme to sufficiently raise PA levels in order to alleviate cardiotoxicity may be investigated in larger studies.

This is the first study using objectively measured PA with an activity tracker to analyse the association between overall PA and markers of myocardial function and myocardial injury. Similarly to previous, larger studies by Foulkes et al. and Antunes et al. in 104 and 93 breast cancer, respectively, which evaluated the effect of an exercise intervention (3x/week aerobic and resistance exercise) on cardiac markers of systolic function between an exercise and usual care group undergoing AC, our study did not find between-group differences for any echocardiographic parameters (15, 23). At AC-end, GLS deteriorated by 1.3% and 1.1% in the exercise groups compared to 1.0% and 0.8% in the usual care groups in the studies by Foulkes et al. and Antunes et al., respectively, with no differences between the groups, despite a significantly greater decline in peak VO_2_ in the usual care groups, a finding that was not confirmed in our study (15, 23).

Against our hypothesis, ET participation during AC did not result in higher PA in the EXduringAC group nor did it lead to a reduced decrease in peak VO_2_ compared to the control group (more details on peak VO_2_ and PA data will be published elsewhere). The mean adjusted decreases in peak VO_2_ at AC-end were −1.7 and −1.6 ml/kg/min in our exercise and usual care group, respectively. This is comparable to the reported changes of −1.5 ml/kg/min of the exercise group and less than decreases of −2.9 ml/kg/ml in the usual care group in the BREXIT study (15), however, losses in peak VO_2_ were greater than changes of 0.9 and −0.9 ml/kg/min in the exercise and usual care group, respectively, found by Antunes et al. (23). The provision of PA-trackers in our cohort may have narrowed the effect of the supervised ET by preventing the drop in VO_2_ in our usual care group, thereby contributing to the smaller between-group differences in our study.

While baseline characteristics in the study by Foulkes et al. were comparable to our cohort (peak VO_2_ 95% and 93% of predicted in the usual care and exercise group, respectively), only 7.5% of patients in the study by Antunes et al. fulfilled the physical activity guidelines at study entry (15, 23), vs. 76% in our population. The lower baseline peak VO_2_ and higher BMI of their patients at albeit similar age suggests that their cohort consisted of less active and less fit patients. In this context, it is important to note that changes in peak VO_2_ are often inversely related to baseline peak VO_2_ (35). In our cohort, there was a significant moderate correlation (*r* = −0.4, *p* = 0.005) between baseline CRF and changes in peak VO_2_ at AC-end (Supplementary Figure 3). The reason for this may be that there is a ceiling effect which allows more potential for improvement in initially less fit patients. Additionally, peak VO_2_ is dependent on motivation and form of the day, where an exceptionally good performance is more likely to be followed by a poorer performance and vice versa, a phenomenon called “regression to the mean” (36, 37). In fact, most studies investigating changes in peak VO_2_ in patients with cancer adjust for baseline values (15, 23, 38, 39). Consequently, the decrease in peak VO_2_ in the exercise groups of the BREXIT and our study at the end of AC, referring to a similar cohort with high baseline peak VO_2_ and the increase in the study by Antunes et al. in less active patients should be interpreted in this context (15, 23). Nevertheless, the decrease in peak VO_2_ at AC-end in our usual care group was similar to the changes reported in the exercise group of the BREXIT study (15), suggesting that a decay in cardiorespiratory fitness was attenuated successfully by the provision of PA trackers by a similar amount as providing exercise training interventions in a comparable cohort.

The fact that MVPA and steps were similar between our study groups allowed the pooled analysis including all patients in a linear model, suggesting that higher levels of MVPA during AC may attenuate myocardial injury. Our findings can be explained by biologically plausible mechanisms and experimentally demonstrated results from animal studies (19). A recent prospective observational study by Peck et al. assessed self-reported MVPA and cardiac function in 88 breast cancer patients over the course of AC- and HER2-therapy (20). In their study, every 30 min increase in MVPA during treatment was associated with a 0.04% improvement in GLS, confirming our results. The dose-response relationship found in the study by Peck and colleagues (20) and our study are suggestive of a causal relationship. Nevertheless, larger studies are needed to confirm our results and provide consistent evidence.

The significant inverse association of hs-Troponin with PA at AC-end in our study is in line with the study by Foulkes and colleagues who found a significantly lesser increase in the exercise group compared to the usual care group (8-fold vs. 16-fold baseline values) (15), or a difference by trend in a smaller study (14), while Antunes et al. did not report between-group differences in hsTnT, opposing our results (23). In contrast to our study, Foulkes et al. did not observe a significant increase in BNP (14), while Antunes et al. found a significant increase in NT-proBNP at AC-end in the usual care group, however this did not lead to differences between the groups (17). There is less evidence on the utility of NT-proBNP or BNP for both, prediction of cancer therapy-induced LV dysfunction (40) and effects of exercise on the increase of this biomarker (19).

Some previous studies, however, have found that ET during AC attenuated the decrease in cardiac function. In the per-protocol analysis by Antunes et al., the decline in GLS was attenuated in the exercise group at 3 months post AC (17). Similarly, a non-randomized CMR study in 27 early breast cancer patients found a significant deterioration in GLS in the control group (1.6 ± 1.4%, *p* = 0.002), whereas GLS was preserved in the exercise group (−0.56 ± 1.5%, *p* = 0.377) (41). Unfortunately, the study did not assess changes in cardiorespiratory fitness (41). In another study of similar size and design (non-RCT), GLS worsened by 0.1% and 0.9% in the exercise and control group, respectively, with no significant difference between groups (14). However, none of the non-randomized studies provided information on total PA, so it can only be assumed that the exercise group had higher levels of PA.

It should be noted, that 2 of the 36 patients with available GLS at baseline and AC-end were male lymphoma patients (6%), who differ in their CVD risk profile compared to female breast cancer patients. However, due to the low number of patients with available primary outcome data, we decided to include these patients in our main analysis and we did not perform a sensitivity analysis.

### 4.1. Strength and limitations

A major strength of our study was the detailed objective assessment of PA during and following AC, which included PA based on accelerometry as well as exercise diary for activities like cycling and swimming. While the Fitbit Zip may overestimate MVPA (42), the between-group comparison of our study is nevertheless valid. Further strengths include the randomized design and use of robust and established primary and secondary endpoints.

The main limitation of this study is the poor compliance with group allocation and exercise programme adherence, with only 72.4% und 58.6% compliant patients according to PP criteria in the EXduringAC and EXpostAC groups, respectively. The adaptation of exercise sessions due to Covid-19 with the consequence that in 4 patients, endurance exercise sessions were shortened or replaced with strength training contributes to the low adherence and is another limitation of the study. Moreover, Covid-19 related adaptations of the centre-based ET-programmes with inclusion of home-based training sessions and differences in supervised ET in the three different centres resulted in poorly standardized centre-based ET. Future studies should therefore carefully plan the implementation and tailored prescription of ET. In this context, it should be noted that Covid-19 restrictions in Switzerland were less strict compared to other European countries which obliged people to remain in their homes. It was therefore feasible for our patients to replace the centre-based training sessions with an outdoor session (i.e., walking or jogging in the forest) and when patients were asked to participate in an online training session via Zoom, the majority preferred to spend time outdoors. In addition, due to recruitment difficulties related to the Covid-19 pandemic, the study was limited by the small sample size (calculated sample size was 102 patients) and patients with available primary outcome data (*n* = 18 in each group), reducing the power of this RCT. We acknowledge that ultrasound-based assessment of GLS is noisy which may hinder the confident analyses of changes in our primary outcome. However, in our clinic, GLS is routinely assessed with ultrasound and we did not change this method to avoid additional assessments for our patients. A further limitation is selection bias, with studies on exercise interventions often resulting in the recruitment of physically active patients. This is confirmed by the fact that our cohort fulfilled the WHO PA guidelines (25) even during AC. Simple use of PA trackers has been shown to increase PA and steps (24, 43), which may explain why our supervised ET intervention did neither lead to a difference in PA nor change in peak VO_2_ between groups. Nevertheless, our study reflects the clinical situation in that private use of PA trackers is common as is poor adherence to scheduled supervised training sessions due to other commitments related to cancer therapies, work and family, or due to fatigue and other, treatment or disease related, side effects. Due to the short time-frame from study inclusion to start of AC, we were not able to perform an objective baseline assessment of PA. We therefore included total MVPA from GPAQ questionnaire to provide an estimate of baseline PA of our study sample (with the limitation that this is a self-reported measure). In addition, the Fitbit Zip only recorded steps/day and minutes exceeding different physical activity thresholds. Therefore, only when the tracker was not worn for a complete day was this day recorded as non-wear time. If trackers were only worn for part of days, the number of steps and activity minutes would have been underestimated in these patients. Further, we did not monitor the wearing location of the tracker.

Additionally, in our PP-analysis, there was a trend for the group × time interaction for GLS at AC-end (*p* = 0.08), suggesting that GLS deteriorated more in the EXduringAC group, opposing our hypothesis. Nevertheless, due to the small sample size, results from this analysis have to be interpreted with caution. However, our study is the third largest RCT to date to report changes in GLS and markers of myocardial injury during AC with and without exercise intervention. In addition, our results are in line with the two largest RCTs who found no difference in GLS in 92 and 73 study patients, despite a between-group difference in peak VO_2_ (15, 23). Nevertheless, decreases in peak VO_2_ in both our groups were comparable to the reported changes in the exercise group of the BREXIT study that implemented a rigorous exercise programme (15). Importantly, we recruited predominantly active patients at low risk for development of cardiotoxicity and it should be noted that the effect of an exercise intervention on markers of myocardial function may be different in patients with more pronounced cardiovascular risk factors and therefore results cannot be generalized to patients at higher risk and or a less active patient population. Future studies should be designed to obtain a better understanding of the needs and physical activity levels of less active patients.

## 5. Conclusion

This is the first study to demonstrate a significant inverse association between objectively measured MVPA and markers of LV dysfunction and myocardial injury.

Objectively measured PA revealed that in physically active patients with cancer, the addition of supervised ET did not result in more PA compared to PA advice and feedback by activity trackers alone. The inclusion of a physically active study population, the relatively low volume and poor standardization of the ET due to Covid-19 and the low compliance with ET contributed to the absence of a difference between our study groups. Nevertheless, the dose-response relationship between PA and cardioprotective effects during AC found in our and previous data strengthens that PA should be recommended to patients undergoing AC. The potential of PA tracking to mitigate cardiotoxicity should be investigated in future, larger studies.

## Data Availability

The raw data supporting the conclusions of this article will be made available by the authors, without undue reservation.

## Lay summary

The study aimed to compare changes in cardiac function, blood markers of damage to the heart muscle and physical fitness between patients performing a supervised exercise training programme during anthracycline chemotherapy and patients receiving only advice on physical activity, with all patients receiving an activity tracker.

• Supervised exercise training did not lead to an increase in physical activity compared to the control intervention of physical activity tracking only and changes in markers of cardiac function, myocardial injury and cardiorespiratory fitness did not differ between the study groups. The amount of physical activity measured by trackers, however, achieved recommended levels in both groups.
• This is the first study that found that the more physical activity patients performed, the smaller the damage to the heart was by the chemotherapy, suggesting that performing sufficient physical activity protects the heart muscle against the risk of damage from anthracycline chemotherapy.
