# Supplementary material for: Supervised exercise training in patients with cancer during anthracycline-based chemotherapy to mitigate cardiotoxicity: a randomized-controlled-trial
Source: Front Cardiovasc Med. 2023 Dec 4;10:1283153. doi: 10.3389/fcvm.2023.1283153 (PMC10725952; doi:10.3389/fcvm.2023.1283153)
Supplement: Supplementary file 1 [file Datasheet1.pdf]

Supplemental Material

Transthoracic echocardiography

Traditional echocardiographic parameters of LV geometry, and systolic and diastolic function were assessed based on contemporary recommendations.(1, 2) LV end-diastolic (LVEDV), and LV end-systolic volumes (LVESV) were calculated using the biplane method. All volumes were indexed for body surface area (BSA). LV systolic function was expressed as ejection fraction (EF), derived from the LVEDV and LVESV. LV diastolic function was assessed by pulse-wave and tissue Doppler in the apical four-chamber view.

Peak systolic LV GLS was assessed using standard 2D apical four-chamber, two-chamber and three-chamber views using speckle-tracking analysis.(3) Manual tracing of the endocardial borders on an end-systolic frame (aortic valve closure) was performed and the myocardial region of interest was adjusted to include all the endocardium and epicardium, excluding the pericardium. Automatic tracing was then applied on subsequent frames. Adequate tracing for each segment was verified and manually corrected, if necessary. GLS was determined by averaging all values of the 18 segments of the three views.

Biomarkers of myocardial injury

Blood samples were obtained from the antecubital vein with the patient in a seated position at each visit preceding the CPET. We measured the plasma concentrations of hsTnT (in ng/L, Roche Elecsys, 2021, three centres), and NT-proBNP (in pg/ml, Roche Elecsys, all centres). One participating centre (5 patients) determined hs-Troponin-I instead of hsTnT. Due to non-comparability of these two biomarkers, only patients with available hsTnT were included in this analysis.

Physical activity tracking and formula

$$\text{Total steps}\left[\frac{\text{steps}}{\text{day}}\right] = \text{steps}\left[\frac{\text{steps}}{\text{day}}\right] + (\text{RPE} * 10 * \text{endurance minutes})$$

$$\text{Moderate to vigorous PA}\left[\frac{\text{min}}{\text{day}}\right] = \text{PA}\left[\frac{\text{min}}{\text{day}}\right] + (\text{endurance minutes with Borg} \geq 12)$$

The formula converting endurance minutes into steps was based on a study by Tudor-Locke who identified the thresholds of 100 steps/min for moderate intensity (3 METs) and 130 steps/min for vigorous intensity (6 METs).(4)RPE according to Borg, on the other hand, is related to METs with ratings of 10-13 on Borg scale corresponding to 3-6 METs.(5)

Of the PA data from Fitbit Zip and training diary, the following parameters were calculated for each patient: daily steps including converted non-step based PA, and daily MVPA including converted non-step based PA.

Serious adverse events

According to the Ordinance on Clinical Trials in Human Research, serious adverse events were defined as any untoward medical occurrence that I)results in death or is life-threatening, ii) requires in-patient hospitalisation or prolongation of existing hospitalisation or III) results in persistent or significant disability or incapacity. Serious adverse events were monitored by an independent data safety monitoring committee of the study and reported to the local ethics committee within 15 days.

References

1. Lang RM, Badano LP, Mor-Avi V, Afilalo J, Armstrong A, Ernande L, et al. Recommendations for cardiac chamber quantification by echocardiography in adults: an update from the American Society of Echocardiography and the European Association of Cardiovascular Imaging. Eur Heart J Cardiovasc Imaging. 2015;16(3):233-70.
2. Nagueh SF, Smiseth OA, Appleton CP, Byrd BF, 3rd, Dokainish H, Edvardsen T, et al. Recommendations for the Evaluation of Left Ventricular Diastolic Function by Echocardiography: An Update from the American Society of Echocardiography and the European Association of Cardiovascular Imaging. Eur Heart J Cardiovasc Imaging. 2016;17(12):1321-60.
3. Voigt JU, Pedrizzetti G, Lysyansky P, Marwick TH, Houle H, Baumann R, et al. Definitions for a common standard for 2D speckle tracking echocardiography: consensus document of the EACVI/ASE/Industry Task Force to standardize deformation imaging. J Am Soc Echocardiogr. 2015;28(2):183-93.
4. Tudor-Locke C, Aguiar EJ, Han H, Ducharme SW, Schuna JM, Jr., Barreira TV, et al. Walking cadence (steps/min) and intensity in 21-40 year olds: CADENCE-adults. The international journal of behavioral nutrition and physical activity. 2019;16(1):8.
5. Norton K, Norton L, Sadgrove D. Position statement on physical activity and exercise intensity terminology. J Sci Med Sport. 2010;13(5):496-502.

Supplement Table 1. Complete list of administered anthracycline-based chemotherapies (AC).

**Breast Cancer**			
Neoadjuvant (with preceding chemotherapy	Pertuzumab/Trastuzumab/ Paclitaxel (or Docetaxel)<br><br>followed by<br><br>Doxorubicin/ Cyclophosphamide	Pembrolizumab/ Carboplatin /Paclitaxel<br><br>followed by<br><br>Pembrolizumab/ Doxorubicin/ Cyclophosphamide	
Adjuvant or neoadjuvant (no preceding chemotherapy)	Doxorubicin/ Cyclophosphamide<br><br>followed by<br><br>Paclitaxel (and in $HER2^+$ patients Trastuzumab)	Doxorubicin/ Cyclophosphamide<br><br>followed by<br><br>Paclitaxel (and in $HER2^+$ patients Pertuzumab/ Trastuzumab)	Epirubicin/ Cyclophosphamide
**Hodgkin's Lymphoma**			
	ABVD:<br><br>Doxorubicin, Bleomycin, Vinblastine, Dacarbazine	Escalated BEACOPP:<br><br>Bleomycin, Etoposide, Doxorubicin, Cyclophosphamide, Vincristin, Procarbazine, Prednisone	
**Non-Hodgkin-Lymphoma**			
	R-CHOP:<br><br>Rituximab, Cyclophosphamide, Doxorubicin, Vincristine, Prednisone	O-CHOP:<br><br>Obinutuzumab,Cyclophosphamide, Doxorubicin, Vincristine, Prednisone	

Supplement Table 2: Comparison of baseline characteristics from patients who discontinued the study (Dropouts) after the first visit with the study cohort (Completers), shown are n (%), median [1st and 3rd quartiles]. Indicated are p-values from Wilcoxon two-sample tests or Fisher's exact test, as appropriate.

**Characteristics**	**Completers (n=51)**	**Dropouts (n=6)**	***p*-value**
Male	3 (5.8)	1 (16.7)	0.367
Age [years]	46 [38, 53]	57 [37, 65]	0.007
Body mass index [kg/m2]	23.9 [21.7, 27.2]	24.6 [22.0, 27.8]	0.594
Systolic blood pressure [mmHg]	115.0 [109.2, 120.0]	117.5 [115, 123.2]	0.240
Diastolic blood pressure [mmHg]	70.0 [65.0, 78.0]	69.0 [65.5, 70.8]	0.493
Hemoglobin [g/L] *	12.9 [11.9, 13.9]	13.5 [12.3, 14.3]	0.801
Cardiorespiratory fitness [ml/min/kg]	25.8 [21.5, 29.9]	21.2 [20.4, 23.1]	0.110
Cardiorespiratory fitness [% of predicted]	103.0 [85.5, 118.0]	93.0 [86.0, 107.0]	0.928
Global longitudinal strain [%]	-21.7 [-22.65, -19.2]	-21.7 [-23.1, -17.5]	0.812
FACT-G score	84.7 [73.1, 93.4]	80.5 [65.0, 92.8]	0.364
FACIT-F score	40.5 [33.0, 46.5]	35.5 [28.3, 45.3]	0.347
**Tumor site**			
Breast	47	5	0.439
Lymphoma	4	1	
**BC disease-stage n=52**			
Stage I	11	1	1
Stage II	27	3	
Stage III	8	1	
Stage IV	1	0	
**BC clinical subtype**			
Hormone-positive (ER+/PR+)	28	2	0.459
HER2-positive	12	2	0.602
Triple negative	12	1	1

Abbreviations: IQR, Interquartile range; BC, Breast cancer; AC, Anthracycline
*Anaemia was defined as hemoglobin level < 120 g/L for women and < 130 g/L for men

Supplement Table 3: Per protocol analysis of changes of echocardiographic parameters and biomarkers of myocardial injury, including patients allocated to the study group with >60% adherence to the exercise programme. Shown are median (1st and 3rd quartiles). Significant p-values for group and time interaction or main effects are indicated and derived from mixed linear models with patients as random factors (intercepts).

	**EXduringAC (n=28)**			**EXpostAC (n=29)**		
	Baseline	Δ baseline to AC-end*	Δ AC-end to follow-up	Baseline	Δ baseline to AC-end*	Δ AC-end to follow-up
GLS [%]	-21.7 (-22.7, -19.2)<br>N=17	1.2 (-1.60, 4.5)<br>N=15	0.2 (-1.80, 1.50)<br>N=17	-21.0 (-22.4, -18.9)<br>N=16	-1.3 (-2.2, 2.5)<br>N=13	0.7 (2.0,3.8)<br>N=12
HR GLS [bpm]	71.0 (63.0, 74.0)<br>N=17	8.0[b] (-0.5, 13.0)<br>N=15	-5.0 (-7.0, 2.0)<br>N=17	68.0 (66.5, 83.0)<br>N=16	7.0[b] (2.0, 10.0)<br>N=13	-4.5 (-12.3, 2.0)<br>N= 12
2D LVEF [%]	62.4 (60.0, 67.9)<br>N= 18	-2.0 (-7.3, 2.9)<br>N= 18	4.1 (-3.6, 6.4)<br>N=19	63.4 (60.0, 65.9)<br>N=17	-2.6 (-4.7, 0.5)<br>N=15	1.8 (-2.6, 3.3)<br>N= 15
LVEDVi [ml/m^2]	51.7 (42.0, 59.6)<br>n=18	0.74 (-5.1, 4.3)<br>N= 18	1.68 (-9.9, 7.1)<br>N=19	48.4 (44.0, 53.3)<br>N=16	-0.80(-11.9, 7.2)<br>N=15	-0.37 (-3.6, 6.6)<br>N= 15
E/*e*'	6.86 (6.30, 8.25)<br>N= 20	-0.19 (-1.58, 1.27)<br>N= 20	0.25 (-0.69, 0.79)<br>N=20	7.20 (5.74, 8.09)<br>N=17	-0.47 (-0.75, 0.97)<br>N=16	0.01 (-0.90, 1.77)<br>N= 15
E/*A* ratio	1.30 (1.09, 1.62)<br>N=20	-0.11 (-0.32, -0.06)<br>N= 20	-0.02 (-0.22, 0.28)<br>N= 20	1.29 (1.04, 1.45)<br>N=17	-0.10 (-0.22, 0.07)<br>N=16	0.04 (-0.27, 0.15)<br>N= 16
LAVI [ml/m^2]	26.7 (22.6, 29.2)[a]<br>N=19	-2.9[c] (-5.0, 0.2)<br>N= 19	1.5 (-3.2, 5.2)<br>N=19	20.3 (18.9, 25.5)<br>N=18	0.9 (-2.5, 2.8)<br>N=14	-1.4 (-4.0, 2.0)<br>N= 13
NT-proBNP [pg/ml]	56.0 (49.0, 99.0)[a]<br>N=19	30.0 (-1.3, 177.0)[b]<br>N=16	-0.5 (-73.5, 18.0)<br>N=18	49.0(49.0, 49.0)<br>N=17	37.0(5.0, 66.0)[b]<br>N=15	-14.5 (-59.8, -10.0)<br>N= 14
hsTnT [ng/L]	4.0 (4.0, 5.0)<br>N=20	7.0 (5.5, 13.5) [b]<br>N= 15	-1.0 (-4.0, 2.0)[b]<br>N=17	4.0 (4.0, 4,0)<br>N=17	11.0 (6.5, 16.0) [b]<br>N=15	-5.5 (-8.8, -2.3)[b]<br>N= 14
hs-C-Reactive Protein [mg/l]	1.1 (0.73, 3.08)<br>N=16	1.0 (0.1, 3.0)<br>N= 16	-0.3 (-1.8, 1.8)<br>N=16	2.39 (0-98, 3.59)<br>N=16	0.8(-0.7, 1.4)<br>N= 15	-1.6 (-2.9, -0.1)<br>N=13
Systolic BP [mmHg]	130.0 (118.5, 140)<br>N=19	-5.0 (-13.0, 10.0)<br>N= 17	-1.0 (-11.0, 4.0)<br>N=17	120.5(112.5, 129.2)<br>N=16	-6.0 (-12.0, 6.0)<br>N=13	-2.0 (-5.5, 7.8)<br>N= 12
Diastolic BP [mmHg]	76.0 (69.0, 84.5)<br>N=19	0.0 (-12.0, 6.0)<br>N=17	-8.0 (-14.0, 12.0)<br>N=17	68.6 (62.8, 80.5)<br>N=16	-2.0 (-10.0, 9.0)<br>N=13	1.5 (-3.0, 10.5)<br>N=12

a, $p < 0.05$ between groups at baseline; b, significant main effect for time; c, significant interaction effect for study group and time; *primary outcome visit. Reference group was ExpostAC.

GLS, global longitudinal strain; HR, heart rate; LVEF, left ventricular ejection fraction; LVEDVi, left-ventricular end-diastolic volume index; hsTnT, high-sensitivity Troponin-T; NT-proBNP, N-terminal pro-brain natriuretic peptide; LAVI, left atrial end-systolic volume index; hs-C-Reactive Protein , high-sensitivity C-Reactive Protein; BP, Blood pressure.

Supplement Table 4: Additional mixed linear models, including group x time interaction and the following predictor variables: studygroup, heart rate to account for changes in preload. Reference category was EXpostAC. Analysis was done on an Intention-to-treat-basis.

	Estimate (95% CI)		t-value	p-value
GLS adjusted				
Intercept	-26.99	(-31.42, -22.57)	-11.83	0.000
EXduringAC	0.15	(-1.53, 1.83)	0.18	0.861
AC-end	0.07	(-1.44, 1.59)	0.10	0.924
Follow-up	0.40	(-1.04, 1.84)	0.54	0.589
HR GLS	0.08	(0.02, 0.15)	2.67	**0.009**
EXduringAC x Visit 2	0.18	(-1.87, 2.24)	0.17	0.863
EXduringAC x Visit 3	1.46	(-0.60, 3.51)	1.38	0.173

Abbreviations: GLS, global longitudinal strain; HR, heart rate; CI, Confidence Interval

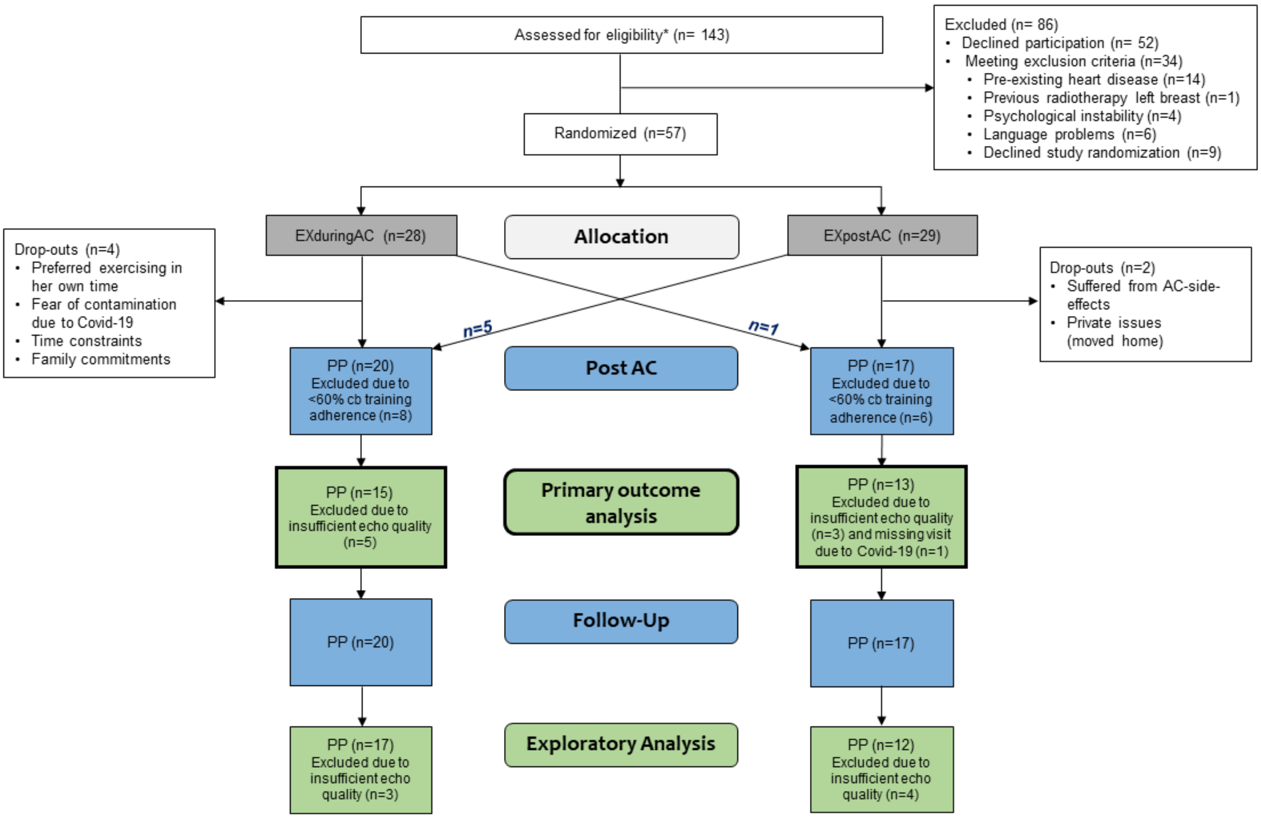

Supplement Figure 1: Study flow illustrating patients included in per-protocol analysis.

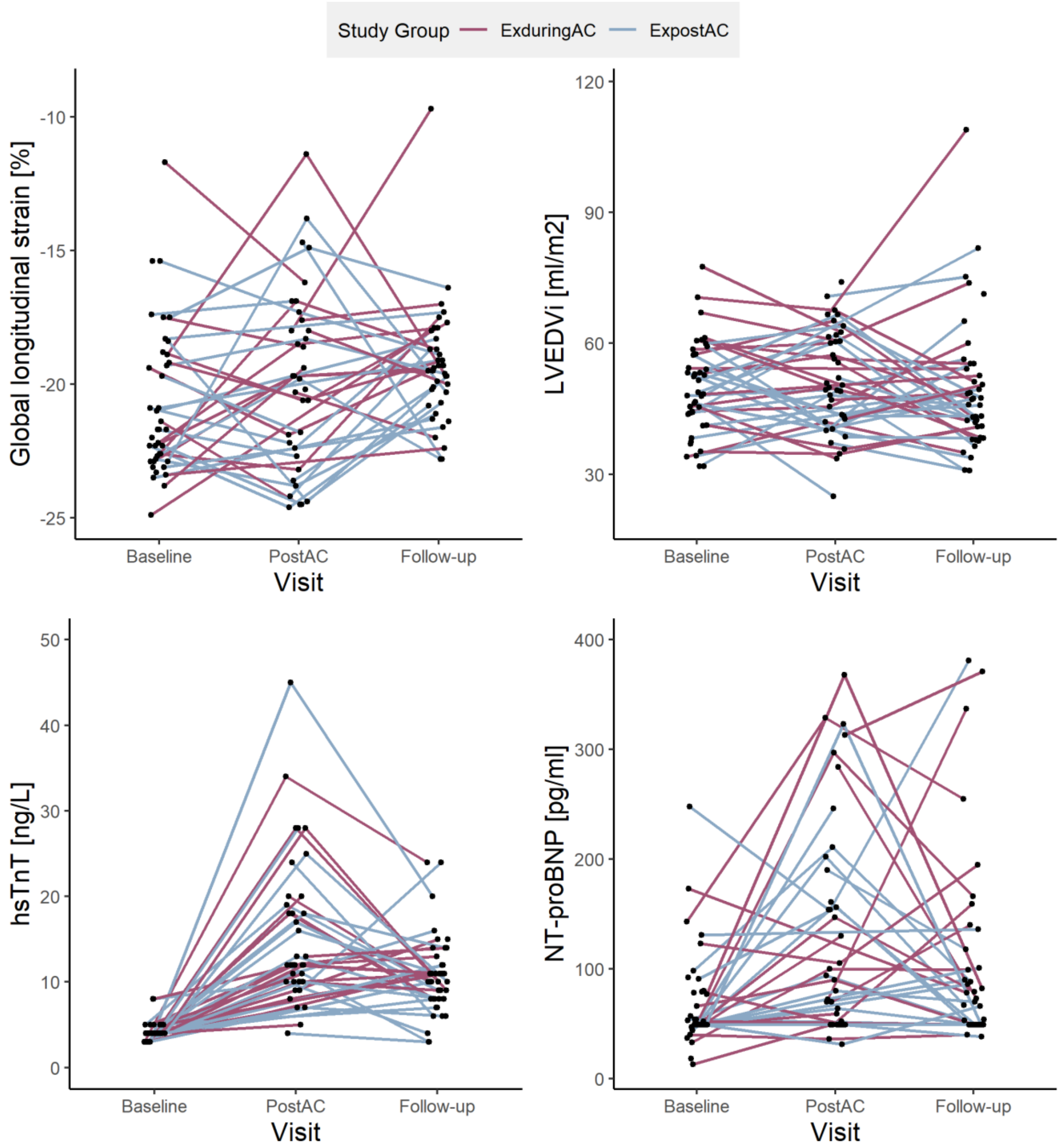

Supplement Figure 2: Lineplots illustrating individual changes in GLS, LVEDVi, hsTnT and NT-proBNP from baseline to AC-end and follow-up.

AC, anthracycline-based chemotherapies; GLS, global longitudinal strain; LVEDVi, left-ventricular end-diastolic volume index; hsTnT, high-sensitivity Troponin-T; NT-proBNP, N-terminal pro-brain natriuretic peptide

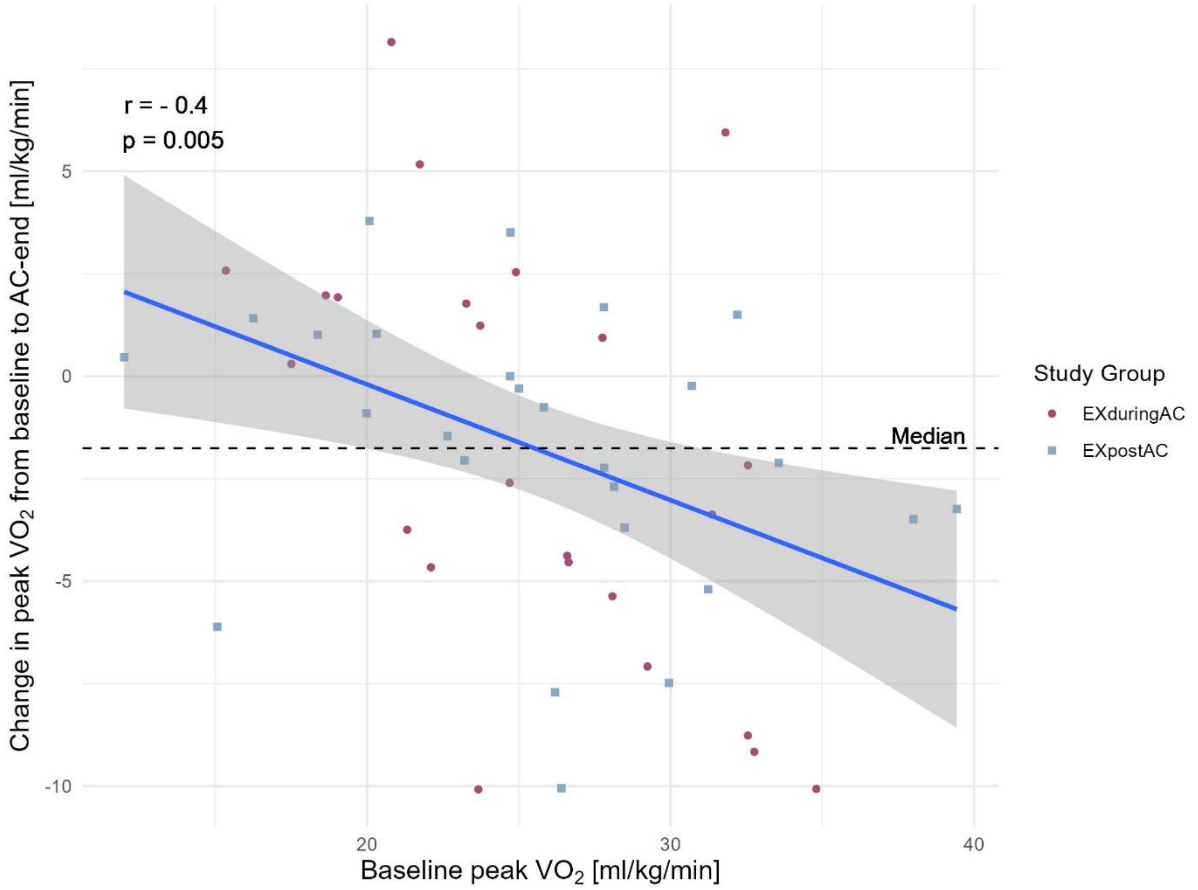

Supplement Figure 3: Change in peak VO_2 from baseline to AC-end, relative to peak VO_2 at baseline. Dashed line represents the median change in peak VO_2 over all patients.

AC, anthracycline-based chemotherapies; VO_2, oxygen uptake.
